# Effect of nutritional intervention on interleukin-18 and alpha-2-macroglobulin in women with obesity and metabolic dysfunction-associated fatty liver disease: a prospective cohort study

**DOI:** 10.1186/s41043-026-01324-8

**Published:** 2026-05-11

**Authors:** Salwa M. El Shebini, Eman R. Youness, Nihad H. Ahmed, Hisham A. Orban, Rehab A. Mohamed, Maha I. A. Moaty

**Affiliations:** 1 Department of Nutrition and Food Sciences, National Research Centre, Dokki, Giza Egypt; 2 Department of Medical Biochemistry, National Research Centre, Dokki, 12622 Giza Egypt

**Keywords:** Metabolic dysfunction-associated fatty liver disease, Obesity, Dietary modification, Interleukin-18, Alpha-2-macroglobulin

## Abstract

**Background:**

Metabolic dysfunction-associated fatty liver disease (MAFLD) is closely linked to metabolic abnormalities, including central obesity, dyslipidaemia, hypertension, hyperglycaemia, and impaired liver function. Identifying effective lifestyle interventions and reliable noninvasive biomarkers remains a public health priority.

**Objective:**

This study aimed to evaluate the effect of dietary modification on metabolic and hepatic parameters in women with MAFLD and to assess the potential role of interleukin-18 (IL-18) and alpha-2-macroglobulin (A2M) as noninvasive biomarkers.

**Methods:**

A prospective interventional study was conducted on 54 women with obesity and at least one criterion of metabolic syndrome. Participants were categorized into mild MAFLD, moderate MAFLD, or healthy liver groups based on ultrasonographic findings. All participants followed a calorie-restricted, balanced diet (1000–1200 kcal/day) for eight weeks. Anthropometric measurements, 24-hour dietary recall, and biochemical parameters, including IL-18, A2M, and liver enzymes, were assessed at baseline and post-intervention.

**Results:**

At baseline, women with moderate MAFLD exhibited significantly higher body mass index (BMI), minimal waist circumference (MWC), low-density lipoprotein cholesterol (LDL-C), and LDL/HDL ratio, along with lower high-density lipoprotein cholesterol (HDL-C), reflecting an adverse metabolic profile. They also reported higher macronutrient intake and lower dietary fiber consumption. A strong positive correlation between IL-18 and A2M (*p* ≤ 0.01) was observed across all groups and was associated with an unfavorable metabolic status. Following the intervention, significant improvements (*p* ≤ 0.05) were observed in weight, BMI, MWC, LDL-C, and gamma-glutamyl transferase (GGT), along with increased HDL-C levels. A highly significant reduction (*p* ≤ 0.01) in IL-18 was observed only among participants with MAFLD, while A2M levels decreased significantly (*p* ≤ 0.05–0.01) across all groups.

**Concluions:**

A calorie-restricted, balanced diet significantly improves metabolic and hepatic parameters in women with MAFLD. These findings highlight the beneficial impact of dietary intervention on liver enzymes and inflammatory markers, particularly GGT, IL-18, and A2M, and support the potential utility of A2M as a noninvasive biomarker and therapeutic target.

## Introduction

Non-alcoholic fatty liver disease (NAFLD), recently reclassified as metabolic dysfunction-associated fatty liver disease (MAFLD), is the most prevalent chronic liver disorder, characterized by excessive fat accumulation in the liver (> 5–10% of liver weight) in the absence of significant alcohol consumption [1, 2]. Globally, NAFLD affects over 30% of adults, with particularly high prevalence in the Middle East and North Africa (MENA) region, where estimates range from 35% to over 40% [3]. Egypt bears one of the highest regional burdens, largely driven by high rates of obesity, insulin resistance and metabolic syndrome [4].

MAFLD is strongly associated with metabolic disorders, including central obesity, dyslipidemia, hypertension, and hyperglycemia, and is closely linked to type 2 diabetes (T2D) [5]. In 2023, the nomenclature was further updated to metabolic dysfunction-associated steatotic liver disease (MASLD) following approval by 70% of the expert panel [6]. MAFLD/MASLD may progress to non-alcoholic steatohepatitis (NASH) or metabolic dysfunction-associated steatohepatitis (MASH), with a significant proportion developing advanced fibrosis [6–8].

Lifestyle factors, particularly excess caloric intake and sedentary behavior, contribute to hepatic lipid accumulation and lipotoxicity, which in turn exacerbate inflammation, oxidative stress, and fibrosis [9]. The liver regulates lipid metabolism through triacylglycerol synthesis, mitochondrial fatty acid oxidation, and very low-density lipoprotein formation. Disruption of these processes may lead to hepatic steatosis and disease progression [10].

Susceptibility to NAFLD/MAFLD and its progression are influenced by genetic variation. Previous studies have identified genes involved in lipid and glucose metabolism, hepatic iron and cholesterol regulation, and epigenetic modifications [11]. Specific polymorphisms, including those in IL-6, IL-1β, and the HFE gene, have been associated with increased severity of steatohepatitis and liver fibrosis. These findings highlight the multifactorial nature of NAFLD/MAFLD and provide a framework for understanding the metabolic and inflammatory mechanisms underlying disease development and progression [12].

Biomarkers, such as acute phase proteins, cytokines, apoptosis and oxidative stress markers, and various miRNAs are used to differentiate the spectrum of NAFLD [13]. Alpha-2-macroglobulin (A2M), an acute phase protein, may protect against fibrosis by inhibiting proteases and modulating hepatic stellate cell activation, thereby limiting excessive collagen accumulation [14–16]. Inflammatory cytokines, particularly interleukins such as IL-18, play key roles in NAFLD progression and have emerged as potential noninvasive biomarkers for identifying individuals at risk of metabolic complications [17, 18].

While previous studies have examined dietary modification in NAFLD or evaluated IL-18 or A2M individually, few have investigated these biomarkers together within a structured dietary intervention [19, 20]. Our study addresses this gap by simultaneously assessing IL-18 and A2M to clarify their interplay in MAFLD progression and their combined response to dietary modification. This dual-marker approach provides mechanistic insight into how nutritional therapy modulates both inflammatory cytokine activity and protease-inhibitor responses, extending beyond traditional single-biomarker or nutrient-based assessments of dietary inflammation.

This study aimed to assess the impact of dietary modification on inflammatory (IL-18) and protease-inhibitor (A2M) biomarkers and to evaluate their combined role in the progression of metabolic dysfunction-associated fatty liver disease (MAFLD). The study focused on middle-aged Egyptian women with obesity, providing population-specific insights to inform targeted prevention and management strategies. Comprehensive biochemical analyses were conducted to assess liver function and biomarker changes, enhancing understanding of the metabolic, inflammatory, and dietary factors involved in MAFLD.

## Subjects and methods

### Study design and participants

This eight-week prospective dietary intervention study was conducted as part of a project funded by the National Research Centre (NRC), Egypt (2022–2024), titled “Dietary Therapy for the Management of Non-Alcoholic Fatty Liver Disease in Overweight and Obese Subjects: New Markers for Early Diagnosis and Follow-Up.” The study protocol was approved by the Ethical Committee of the NRC in accordance with the Declaration of Helsinki (Approval No. 13050204-2), and a written informed consent was obtained from each participant after a full explanation of the study objectives.

Participants were recruited from NRC employees using a convenience sampling approach. A total of 70 adult women with obesity and features of metabolic syndrome (MetS) were initially enrolled, of whom 54 completed the study. MetS was defined according to the International Diabetes Federation (IDF) criteria, including central obesity (waist circumference ≥ 80 cm for women), elevated triglycerides, reduced high-density lipoprotein cholesterol, hypertension, and elevated fasting plasma glucose [21, 22].

The eight-week intervention period was selected based on previous evidence demonstrating that meaningful metabolic and inflammatory improvements in MAFLD can be achieved within this timeframe [23, 24]. Only female participants were included to minimize sex-related biological variability and due to the higher prevalence of obesity and better adherence to dietary interventions among women in the studied Egyptian population [25]. All participants were highly educated (at least a university-level education), NRC employees from a middle socioeconomic background.

Liver steatosis was assessed using ultrasonography [26], a recommended screening tool for suspected MAFLD according to the European Association for the Study of the Liver (EASL) guidelines [27]. Participants were categorized into three groups based on liver status: healthy liver (*n* = 22), mild MAFLD (*n* = 18), and moderate MAFLD (*n* = 14).

For consistency, the term MAFLD was used throughout this study, as it aligns with the applied diagnostic criteria based on metabolic dysfunction. The terms NAFLD and MASLD were used only when referring to previously published studies to reflect the evolving nomenclature [7, 28].

### Intervention

All participants followed a calorie-restricted, balanced diet based on the Dietary Approaches to Stop Hypertension (DASH) pattern, with an energy intake of 1000–1200 kcal/day. The nutritional intervention included a defined macronutrient composition, and caloric intake was individualized to achieve a hypocaloric regimen tailored to each participant. Dietary guidance was provided through structured counseling sessions supported by written materials, including meal plans and detailed instructions. Participants were monitored through weekly individual follow-up sessions conducted by dietitians and research staff to assess adherence, address questions, and record body weight.

### Outcome measures

Outcome assessments included anthropometric measurements, dietary intake (assessed by 24-hour dietary recall), and biochemical parameters. Particular emphasis was placed on interleukin-18 (IL-18) and alpha-2-macroglobulin (A2M) to evaluate metabolic and hepatic responses to the dietary intervention.

### Sample size and power

The sample size was determined by the number of eligible participants who met the inclusion criteria and agreed to participate, consistent with the exploratory nature of the study. A post hoc power analysis using G*Power software (version 3.1) indicated that the final sample size (*n* = 54) was sufficient to detect meaningful differences among the three groups, assuming a moderate effect size (f = 0.30), a two-sided α of 0.05, and 80% power, in line with previous exploratory studies in NAFLD/MAFLD populations [29, 30].

### Inclusion and exclusion criteria

Participants were eligible if they were women aged 30–60 years with obesity (body mass index ≥ 30 kg/m²) and a diagnosis of MAFLD confirmed by hepatic ultrasonography, along with at least one component of metabolic syndrome (e.g., hyperglycaemia, hypertension, dyslipidaemia, or central obesity). Additional criteria included stable body weight (± 3 kg) over the previous three months, willingness to adhere to the dietary protocol and follow-up schedule, and the ability to provide informed consent.

Participants were excluded if they had liver diseases other than MAFLD, type 1 diabetes or poorly controlled type 2 diabetes (HbA1c > 9%). Additional criteria comprised severe cardiovascular, renal, endocrine or systemic inflammatory diseases, as well as malignancy. Pregnancy or breastfeeding, use of medications affecting liver function or inflammatory pathways, recent participation in another clinical or dietary study, or inability to comply with the study protocol were further grounds for exclusion.

### Methodology

All participants underwent clinical assessment, including medical history, in addition to the following investigations:

### Anthropometric parameters and blood pressure measurements

The relevant anthropometric parameters were documented according to the methodology established by Sebo et al. [31]. These parameters include height, weight and minimal waist circumference (MWC). The BMI was calculated using the formula weight in kilograms divided by height in meters squared. Blood pressure (BP) measurements for each patient were taken three times, and the average value was recorded. To ensure accuracy, all measurements were conducted by the same researcher.

### Blood collection and biochemical assessments

Fasting blood samples were collected from patients after a 12-hour fasting period. The samples were permitted to clot at room temperature, followed by centrifugation to separate the sera. Fasting blood glucose (FBG**)** levels were measured on fresh samples utilizing the glucose oxidase method [32]. Other biochemical parameters were analyzed using fasting sera that had been stored at −70 °C until required.

Lipid profiles in the form of fasting triglycerides (TG), LDL-C and HDL-C were assessed using enzymatic method according to the instructions supplied in the kits: triglycerides proceed No 2100 [33], LDL C proceed No 5500 Stan Bio Liqui color [34] and HDL-C proceed No 0599 Stan Bio Liqui color [35], respectively. Very low-density lipoprotein cholesterol (VLDL-C) as TG/5, and the risk ratio: LDL/HDL were calculated.

Serum level of GGT was measured using standard enzymatic colorimetric methods, which are widely applied in clinical biochemistry [36]. Fasting serum IL-18 and A2M concentrations were quantified using a commercially available enzyme-linked immunosorbent assay (ELISA) kit supplied by INNOVA BIOTEC CO-LTD CATA. No. In-Hu2760 following the manufacturers’ protocols [37, 38] respectively.

### Dietary recalls

Dietary intake was evaluated using 24-hour dietary recalls collected over three consecutive days, including one weekend day by self-reporting. Participants provided detailed information on all foods and beverages consumed, with portion sizes estimated using household measures, food models, or photographic aids. Nutrient intakes were analyzed using the NutriSurvey (2007) nutrition analysis software [39]. This method has been widely used and validated for the assessment of macronutrient and fiber intake [40–42]. In addition, a semi-quantitative food frequency questionnaire (FFQ) adapted to Egyptian diets was reported [43]. The habitual dietary intake of the volunteers and the prescribed regimen was analyzed in comparison to the DASH dietary pattern, and a score was assigned to each diet based on Gunther DASH Index evaluation [44].

### Radiological examination of the liver

**Liver imaging** was performed with abdominal ultrasonography [26], an accurate and non-invasive diagnostic test, on a different day after a 6-hour fast in order to avoid gas in the bowel and thereby enhance liver visualization. The study was performed by an experienced sonographer who was blinded to all aspects of the clinical history. Imaging was performed using the Siemens ACUSON S3000 Ultrasound-HFLX system using a 6C1 HD curved-array transducer and Acoustic Radiation Force Impulse (ARFI) technology (Virtual Touch Imaging and Virtual Touch Quantification). This scanning and storage system permitted both qualitative and quantitative assessments of liver stiffness and allowed accurate grading of hepatic steatosis. Liver stiffness measurements were obtained from seven hepatic segments and only findings related to steatosis were reported. Cases data indicating coarse parenchyma, fibrosis, focal lesions or fat-sparing areas were not included in study to focus the assessment strictly to staging fatty liver.

### Statistical evaluation

All statistical analyses were performed using SPSS software (Chicago, IL, USA), version 26.0. The normality of continuous variables was assessed using the Shapiro-Wilk test, supported by visual inspection of histograms and Q-Q plots. As the data were normally distributed, parametric tests were used. Results are expressed as mean ± standard error of the mean (SEM). Between-group differences were analyzed using one-way ANOVA followed by appropriate post hoc tests. Paired-sample *t*-tests were applied to evaluate within-group changes across the two phases. Correlations between variables were examined using Pearson’s correlation coefficient. A p-value ≤ 0.05 was considered statistically significant.

## Results

At baseline, women with MAFLD, particularly those with moderate fatty liver, were older and exhibited significantly higher body weight (94.95 ± 2.92 vs. 81.83 ± 3.83 and 81.01 ± 3.02 kg, *p* ≤ 0.01), BMI (38.26 ± 1.16 vs. 33.64 ± 1.19 and 34.01 ± 1.09 kg/m², *p* ≤ 0.05), and MWC (97.00 ± 1.61 vs. 88.33 ± 3.01 and 88.81 ± 2.08 cm, *p* ≤ 0.01) compared with the healthy and mild MAFLD groups, respectively. No significant differences were observed in systolic or diastolic blood pressure among the groups at baseline.

Following the nutritional intervention, all groups showed significant reductions in anthropometric measures (*p* ≤ 0.01), while a significant decrease in diastolic blood pressure was observed only in the moderate MAFLD group (Table 1).

**Table 1:** Comparisons of age, anthropometric measurements, and blood pressure values among the studied groups at both the initial and final visits

Parameters		Healthy liver (no.=22)	Mild fatty liver (no.=18)	Moderate fatty Liver (no.=14)
		Mean ± SE		
**Age (years)**		43.89 ± 2.26^a^	50.70 ± 2.20^b^	59.13 ± 1.46^c^
**Height (cm)**		155.56 ± 1.45^a^	154.25 ± 1.56^a^	157.60 ± 0.96^a^
**Weight** **(kg)**	Initial	81.83 ± 3.83^a^	81.01 ± 3.02^a^	94.95 ± 2.92^b^
	Final	79.06 ± 3.91^**^^d^	78.94 ± 3.58^**^^d^	93.13 ± 2.86^**^^e^
**BMI** **(kg/m** ^**2**^**)**	Initial	33.64 ± 1.19^a^	34.01 ± 1.09^a^	38.26 ± 1.16^b^
	Final	32.48 ± 1.23^**^^d^	33.32 ± 1.12^**^^d^	37.52 ± 1.12^**^^e^
**MWC** **(cm)**	Initial	88.33 ± 3.01^a^	88.81 ± 2.08^a^	97.00 ± 1.61^b^
	Final	87.06 ± 2.97^**^^d^	85.57 ± 3.99^**^^d^	94.70 ± 1.54^**^^e^
**SBP** **(mmHg)**	Initial	122.89 ± 3.03^a^	122.00 ± 4.51^a^	124.90 ± 2.36^a^
	Final	119.00 ± 2.02^*^^d^	120.14 ± 3.55^d^	122.22 ± 4.01^d^
**DBP (mmHg)**	Initial	80.67 ± 2.37^a^	76.25 ± 2.54^a^	79.20 ± 0.97^a^
	Final	79.67 ± 2.16^d^	76.57 ± 3.67^d, e^	73.60 ± 1.83^*^^e^

BMI: Body Mass Index, MWC: Minimal Waist Circumference, SBP: Systolic Blood Pressure, DBP: Diastolic Blood Pressure

Data are presented as mean ± SEM. One-way ANOVA was used to compare between groups, and paired t-test was used to compare between the initial and final visits within the same group

Superscript letters (a, b, c, d, e) indicate significant differences between groups; groups that do not share the same letters differ significantly (p≤ 0.05). Asterisks denote within-group pre-post changes: *p ≤ 0.05, **p ≤ 0.01

**Table 2:** The comparison of total caloric and macronutrient intake among the three studied groups, both in their habitual diet and the prescribed dietary regimen, with different percent

Nutrients	Habitual diet			Regimen	RDAs	F
	Healthy liver (*n* = 22)	Mild fatty liver (*n* = 18)	Moderate fatty liver (*n* = 14)			
	Mean ± SE % Of RDAs for total calories, fibers& cholesterol					
**Total Calories (kcal)**	1976.18 ± 41.15 89.04%^* a^	2156.91 ± 45.08 98.04%^* b^	2412.80 ± 36.24 109.67%^** c^	1060.27 ± 20.12 48.19%	2200	0.021*
**Cholesterol (g)**	194.25 ± 11.82 97.26^** a^	208.52 ± 10.48 104.26^** b^	236.03 ± 5.93 118.02%^** c^	84.02 ± 20.11 42.01%	200	0.056
**Fiber/1000 calories (g)**	11.59 ± 2.13 92.72%	9.28 ± 1.98 74.24%^* b^	7.16 ± 1.42 57.28%^** c^	11.78 ± 2.03 94.24%	12.5	0.003**

	Mean ± SE % Of Total carioles intake form (protein, total fat, carbohydrates &SFAs)					
**Protein(g)**	79.16 ± 6.42 16.02%	74.01 ± 3.91 13.73%	82.46 ± 7.30 13.67%	45.75 ± 8.43 17.24%	50	0.064
**Carbohydrates (g)**	210.80 ± 9.18 42.67%^* a^	244.74 ± 12.94 45.39%^* b^	259.02 ± 12.28 42.91%^* c^	142.30 ± 15.02 53.72%	300	0.044*
**Total fat (g)**	90.71 ± 5.44 41.32% ^* a^	97.99 ± 3.35 40.89%^* b^	116.32 ± 6.46 43.39%^*c^	34.23 ± 6.85 29.06%	60	0.047*
**SFAs(g)**	24.59 ± 1.45 11.20%^** a^	30.79 ± 1.71 12.85%^** b^	34.94 ± 1.29 13.03%^** c^	7.40 ± 0.10 6.23%	Not more than 7% of Total Calorie	0.162

SFAs: Saturated fatty acids. RDA (Recommended Daily Allowance). Source: WHO/FAO [45]

*Significant at p ≤ 0.05, ** Highly Significant at P≤ 0.001, a; Regimen vs. Healthy Liver, b; Regimen vs. Mild Fatty Liver, c; Regimen vs. Moderate Fatty Liver. F significant differences between all groups*≤0.05 **≤0.01

Notably the moderate MAFLD group consumed significantly more total calories, fat, and cholesterol, and less fiber, than the other groups with p-values ranging from ≤ 0.05 to ≤ 0.01. A comparison of nutrient intake from the described regimen revealed significant differences when contrasted with the habitual diet consumed by the three groups (Table 2). The recommended daily allowance (RDA) values were based on the World Health Organization (WHO) and the Food and Agriculture Organization of the United Nations (FAO) [45].

Dietary intake scores varied across the studied groups, with women with mild and moderate MAFLD showing the lowest adherence to the DASH diet (29.35 and 19.71 out of 80, respectively). In contrast, women with healthy livers demonstrated higher adherence (44.12 out of 80), while the prescribed dietary regimen achieved the highest score (60.56 out of 80). These differences were statistically significant (*p* ≤ 0.001) (Table 3).

**Table 3:** Scores of the habitual dietary intake of the different studied groups and the recommended regimen according to Günther’s DASH index

Component	Foods	Standard for maximum score	Habitual diet			Regimen	F
			Healthy liver (*n* = 22)	Mild fatty liver (*n* = 18)	Moderate fatty liver (*n* = 14)		
**Grains**	Total 5 High fiber5	≥ 6 servings/day ≥ 50% of daily servings	6.73 ± 0.23	4.89 ± 0.17* b	2.57 ± 0.35** c	8.88 ± 0.14	0.005
**Vegetables**	Includes potatoes10	≥ 4 servings per day	5.82 ± 0.15* a	4.01 ± 0.19* b	2.01 ± 0.29** c	8.98 ± 0.12	0.003
**Fruits**	All Fruits& Fruits Juice10	≥ 4 servings per day	6.01 ± 0.28* a	3.33 ± 0.21* b	1.01 ± 0.20** c	8.92 ± 0.20	0.001
**Milk and Dairy products**	Total 5 Low fat 5	≥ 2 servings/day ≥ 75% of daily serving	3.45 ± 0.22	4.78 ± 0.23	5.43 ± 0.22	4.98 ± 0.19	0.120
**Meat**,** poultry**,** fish**,** eggs**	10	≤ 2 servings per day	6.02 ± 0.28	4.44 ± 0.22* b	4.14 ± 0.31* c	7.89 ± 0.15	0.022
**Nuts**,** seeds**,** legumes**	10	≥ 4 servings per week	6.18 ± 0.21	3.11 ± 0.18** b	1.29 ± 0.26** c	8.99 ± 0.19	0.001
**Fat**,** Oil**	10	≤ 3 servings per day	4.80 ± 0.24	2.29 ± 0.06** b	2.03 ± 0.02** c	6.90 ± 0.21	0.002
**Sweet**	10	≤ 5 servings per week	5.11 ± 0.28	2.50 ± 0.013** b	1.23 ± 0.01** c	5.02 ± 0.15	0.001
**Total**	80		44.12* a	29.35**b	19.71** c	60.56	0.001

*Significant at *P* ≤ 0.05, ** Highly Significant at *P* ≤ 0.001

a; Regimen Vs Healthy Liver, b; Regimen Vs Mild Fatty Liver, c; Regimen Vs Moderate Fatty Liver

F values: differences between the 4 groups including the regimen, significant *≤0.05** Highly Significant at ≤ 0.001

Prior to the dietary intervention, the findings indicated a minor elevation in FBG levels across the three groups prior to the dietary intervention, although these differences were not statistically significant. In contrast, TG levels and VLDL-C were notably higher in patients with MAFLD, with significant differences observed when compared to the healthy group. The serum concentration of LDL-C was elevated in all three groups, with the highest levels found in patients with moderate fatty liver, showing a significant difference. The levels of HDL-C were low, particularly in the moderate fatty liver group, where the lowest concentration was recorded, and this difference was significant. The mean serum GGT levels remained within the normal range across all groups, but significant differences were noted between moderate fatty liver patients and the healthy group. No significant differences were observed in the mean serum concentrations of IL-18 and A2M among the three groups (Table 4).

Following the dietary intervention, the average serum concentration of FBG showed a numerical decline in the healthy liver group, and significant declines in both the MAFLD groups. A notable reduction in the mean levels of TG, VLDL-C, and IL-18 was observed exclusively in patients with fatty liver. In contrast, LDL-C and GGT exhibited significant reductions in all groups. Additionally, HDL-C levels significantly increased across all groups. Regarding A2M concentrations, a significant decrease was identified across the three groups at the P-values 0.05 − 0.01 (Table 4).

Significant correlations were observed between IL-18 and A2M across all studied groups, with a highly significant positive association (*p* ≤ 0.01).

For IL-18, women with healthy livers showed significant positive correlations with BMI, VLDL-C, GGT, and total carbohydrate intake (*p* ≤ 0.05). In the mild MAFLD group, IL-18 was negatively correlated with HDL-C (*p* ≤ 0.01) and dietary fiber (*p* ≤ 0.05), and positively correlated with saturated fatty acids (SFAs) (*p* ≤ 0.05). In the moderate MAFLD group, significant positive correlations were observed with VLDL-C (*p* ≤ 0.05) and GGT (*p* ≤ 0.01), along with a negative correlation with protein intake and a positive correlation with total fat intake (*p* ≤ 0.05).

Regarding A2M, women with healthy livers showed a significant positive correlation only with total carbohydrate intake (*p* ≤ 0.05). In the mild MAFLD group, A2M was positively correlated with BMI and MWC (*p* ≤ 0.05), negatively correlated with HDL-C (*p* ≤ 0.01) and dietary fiber (*p* ≤ 0.05), and positively correlated with SFAs (*p* ≤ 0.01). In the moderate MAFLD group, A2M showed positive correlations with age and GGT, and a negative correlation with HDL-C (*p* ≤ 0.05). Additionally, significant negative correlations were observed with protein intake (*p* ≤ 0.01) and fiber (*p* ≤ 0.05), while positive correlations were found with total fat intake (*p* ≤ 0.01) and SFAs (*p* ≤ 0.05) (Table 5).

**Table 4:** Comparison between the mean values of the biochemical markers of the studied groups (total no. 54 subjects) at the initial and final visits

Parameters		Healthy liver (no.=22)	Mild NAFLD (no.=18)	Moderate NAFLD (no.=14)
**FBG (mg/dL)**	Initial	110.92 ± 10.31^a^	103.38 ± 3.97^a^	106.75 ± 5.62^a^
	Final	107.44 ± 8.58^d^	98.36 ± 3.79^*^^d^	101.75 ± 3.59^*^^d^
**TG (mg/dL)**	Initial	108.86 ± 6.05^a^	120.75 ± 3.36^a, b^	131.00 ± 4.26
	Final	98.86 ± 1.37^d^	109.38 ± 1.97^**^^e^	109.63 ± 3.26^**^^e^
**LDL-C (mg/dL)**	Initial	191.71 ± 11.98^a^	186.13 ± 3.83^a^	203.12 ± 4.04^b^
	Final	184.57 ± 9.50^**^^d, e^	174.00 ± 3.49^**^^d^	195.38 ± 3.08^**^^e^
**VLDL-C** **(mg/dL)**	Initial	21.77 ± 1.21^a^	24.15 ± 0.67^a, b^	26.20 ± 0.85^b^
	Final	19.77 ± 0.27^d^	21.87 ± 0.39^**^^e^	21.93±0.65^**^^e^
**HDL-C** **(mg/dL)**	Initial	45.67 ± 2.56^a^	49.13 ± 1.35^a^	40.50 ± 0.99^b^
	Final	55.00 ± 2.79^**^^d^	66.63 ± 0.68^**^^e^	54.50 ± 2.58^**^^d^
**Risk factor** **(LDL/HDL)**	Initial	4.04 ± 0.48^a, b^	3.93 ± 0.17^a^	4.88 ± 0.20^b^
	Final	3.07 ± 0.19*^a^	2.58 ± 0.05^**^^b^	3.31 ± 0.10^**^^a^
**GGT (U/L)**	Initial	14.91 ± 0.21^a^	15.78±0.0.47^a, b^	16.09 ± 0.39^b^
	Final	13.55±0.43**^d^	15.28±0.51^**^^e^	14.67±0.0.63^**^^d, e^
**IL-18 (pg/mL)**	Initial	87.77 ± 3.32^a^	96.79 ± 3.54^a^	98.02 ± 8.23^a^
	Final	84.26 ± 2.89^d^	85.25 ± 4.12^**^^d^	87.92 ± 6.11^**^^d^
**A2M** **(ng/mL)**	Initial	10.67 ± 0.45^a^	11.97 ± 0.27^a^	11.89 ± 1.08^a^
	Final	9.82±0.0.45^**^^d^	10.62 ± 0.33^**^^d^	11.03 ± 1.18^*^^d^

FBG: Fasting Blood Glucose, TG: Triglycerides, LDL-C: Low Density Lipoprotein Cholesterol, VLDL-C: Very Low-Density Lipoprotein Cholesterol, HDL-C: High-density Lipoprotein Cholesterol, GGT: Gamma Glutamyl Transferase, IL-18: Interleukin-18, A2M: Alpha 2 Macroglobulin

Values are presented as mean ± SEM. Superscript letters (a, b, c, d, e) indicate significant differences between groups; groups that do not share the same letters differ significantly (p ≤ 0.05). Asterisks denote within-group pre-post changes: *p ≤ 0.05, **p ≤ 0.01

**Table 5:** Correlation coefficients between IL-18, A2M, and relevant anthropometric, biochemical, and habitual dietary parameters at the initial visit among the three study groups

Parameters	IL-18			A2M		
	Healthy liver (no.=22)	Mild fatty liver (no.=18)	Moderate fatty liver (no.=14)	Healthy liver (no.=22)	Mild fatty liver (no.=18)	Moderate fatty liver (no.=14)
**Age (years)**	N.S.	N.S.	N.S.	N.S.	N.S.	**0.532** ^*^
**BMI (kg/m** ^**2**^ **)**	**0.512** ^*^	N.S.	N.S.	N.S.	**0.500** ^*^	N.S.
**MWC (cm)**	N.S.	N.S.	N.S.	N.S.	**0.616** ^*^	. N.S.
**Total Calories (Kcal)**	N.S.	N.S.	N.S.	N.S.	N.S.	N.S.
**T. Carbohydrates(g)**	**0.492** ^*^	N.S.	N.S.	**0.528** ^*^	N.S.	N.S.
**Fiber (g)**	N.S.	**− 0.586** ^*^	N.S.	N.S.	**− 0.532** ^*^	**− 0.496** ^*^
**Total Protein (g)**	N.S.	N.S.	**− 0.597** ^*^	N.S.	N.S.	**− 0.684** ^**^
**Total Fat (g)**	N.S.	N.S.	**0.541** ^*^	N.S.	N.S.	**0.615** ^**^
**SFAs (g)**	N.S.	**0.605** ^*^	N.S.	N.S.	**0.837** ^**^	**0.528** ^*^
**FBG (mg/dL)**	N.S.	N.S.	N.S.	N.S.	N.S.	N.S.
**HDL-C (mg/dL)**	N.S.	**− 0.721** ^**^	N.S.	N.S.	**− 0.822** ^**^	**− 0.598** ^*^
**VLDL-C (mg/dL)**	**0.584** ^*^	N.S.	**0.557** ^*^	N.S.	N.S.	N.S.
**GGT (U/L)**	**0.578** ^*^	N.S.	**0.669** ^**^	N.S.	N.S.	**0.616** ^*^
**IL-18 (pg/mL)**	1	1	1	**0.662** ^**^	**0.697** ^**^	**0.946** ^**^
**A2M (ng/mL)**	**0.662** ^**^	**0.697** ^**^	**0.946** ^**^	1	1	1

BMI: Body Mass Index, MWC: Minimal Waist Circumference, SFA: Saturated fatty acids, FBG: Fasting Blood Glucose, HDL-C: High-Density Lipoprotein Cholesterol, VLDL-C: Very Low-Density Lipoprotein Cholesterol, GGT: gamma glutamyl transferase, IL-18: Interleukin-18, A2M: Alpha-2- Macroglobulin

Numbers presented in this table are the value of r =correlation coefficient. Bold values indicate statistically significant results

**. Correlation is significant at the 0.01 level (2-tailed). *. Correlation is significant at the 0.05 level (2-tailed)

**Fig. 1:**
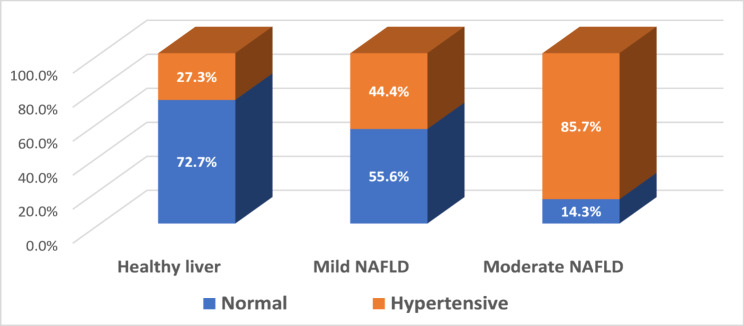
percentage of women suffering from hypertension among the three groups

Figure 1 illustrates the percentage of women experiencing hypertension across the three groups. The data indicated that 27.3%, 44.4% and 85.7% of the women in the three groups respectively were affected by hypertension, with the highest percentage observed in those with moderate fatty liver disease.

**Fig. 2:**
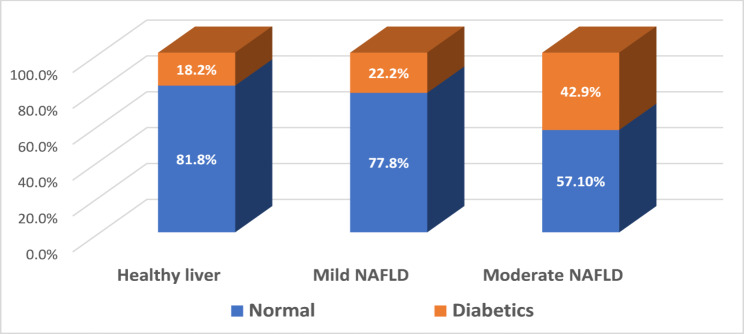
percentage of women suffering from diabetes mellitus among the three groups

Figure 2 illustrates the proportion of women diagnosed with diabetes mellitus across the three groups. The findings indicated that the highest percentage was observed in patients with moderate fatty liver (42.9%), whereas the lowest percentage was recorded in the healthy liver group (18.2%).

The diabetic and hypertensive subjects were on medication and well-controlled, such that their fasting blood sugar and blood pressure measurements were within the normal range.

## Discussion

Non-alcoholic fatty liver disease (NAFLD) is a chronic progressive liver condition resulting from over-nutrition and insulin resistance (IR) in genetically susceptible individuals. The spectrum of NAFLD ranges from non-alcoholic fatty liver and non-alcoholic steatohepatitis (NASH) to progressive fibrosis, cirrhosis, and hepatocellular carcinoma (HCC) [7].

This study found that individuals with MAFLD had increased BMI, waist circumference, LDL-C, and risk factors, along with low HDL-C and poor dietary habits characterized by high macronutrient intake and low fiber consumption. Positive correlations between IL-18, A2M, and GGT were observed in moderate fatty liver cases. Dietary intervention significantly improved biochemical parameters and promoted weight loss, highlighting the importance of a healthy balanced diet in managing MASLD and supporting the role of these biomarkers in disease progression.

### Metabolic comorbidities and disease severity

Our findings demonstrate a clear association between the severity of hepatic steatosis and the prevalence of metabolic comorbidities. Hypertension and type 2 diabetes mellitus were markedly more frequent in patients with moderate steatosis compared with those with mild disease or healthy livers, despite most patients being under pharmacological treatment. These observations align with previous reports indicating that the coexistence of multiple metabolic abnormalities accelerates NAFLD progression and worsens clinical outcomes [5].

### Obesity patterns and anthropometric indicators

Anthropometric assessment observed in the studied sample revealed significantly elevated BMI and MWC, particularly among patients with moderate fatty liver. Abdominal obesity, reflected by a MWC exceeding 88 cm, is a diagnostic criterion for metabolic syndrome and has been strongly linked to NAFLD [46]. Data from NHANES and other studies have shown that compound obesity, characterized by both increased BMI and central adiposity, confers the highest risk for NAFLD [47], supporting our findings and reinforcing the role of visceral fat in disease pathogenesis.

### Dyslipidemia and MAFLD

Atherogenic dyslipidemia is commonly found in a significant number of individuals with NAFLD, arising from both hepatic and peripheral insulin resistance, as well as changes in hepatic glucose and lipoprotein metabolism, gut dysbiosis, and genetic influences [48, 49]. Biochemical analysis of the current study revealed alteration in the lipid profile, marked by increased levels of LDL-C and the risk factor (LDL/HDL), and decreased levels of HDL-C especially in patients with moderate MAFLD. While TG and VLDL-C levels remained within the normal range, however they were notably higher in patients with moderate fatty liver. Dyslipidemia is recognized as a contributing factor to NAFLD. Mansour-Ghanaei and his colleges [50], reported individuals diagnosed with NAFLD exhibited elevated TC, higher LDL/HDL and TC/HDL ratios, alongside reduced HDL levels when compared to those without NAFLD [42]. Additionally, a notable correlation was found between TG and NAFLD, although no significant association was identified between LDL and NAFLD. In a study by Santhoshakumari et al. [51], patients with NAFLD demonstrated increased levels of TC, LDL, and TG, while their HDL levels were lower compared to the control group, indicating a significantly higher prevalence of dyslipidemia in the NAFLD cohort [51]. Another investigation revealed that the average LDL and TC levels among NAFLD patients exceeded the normal range [52]. Furthermore, Novakovic et al. [53] conducted a study in Serbia that compared biochemical parameters in relation to NAFLD, revealing significant associations between TG, LDL, TC, and HDL, along with an inverse relationship with HDL levels. Similar findings were reported in studies by Pardhe et al. [54].

### Biomarkers of liver injury: GGT, IL-18, and A2M

The challenges in evaluating the prevalence and diagnosis of NAFLD stem from the lack of early signs and symptoms, the limited sensitivity of liver enzymes in detecting the condition, and the necessity of performing a liver biopsy as the gold standard for diagnosis, despite its drawbacks. Non-invasive techniques utilize various methods, such as imaging modalities like ultrasound, transient elastography, computed tomography, and magnetic resonance imaging (MRI), which can identify simple hepatic steatosis but are unable to differentiate non-alcoholic steatohepatitis. In this context, there are biomarkers that can distinguish the disease spectrum, including acute phase proteins, cytokines, apoptosis markers, and oxidative stress indicators, along with a range of miRNAs associated with NAFLD that may serve as blood biomarkers for progressive liver damage [14]. Data of this study demonstrated that the mean serum of GGT levels remained within the normal range across all groups, but significant differences were noted between fatty liver patients and the healthy group. In a 2023 study revealed that baseline GGT levels are associated with enhancements in the FibroScan-aspartate aminotransferase (FAST) score after pemafibrate treatment in NAFLD patients suffering from dyslipidemia. Notably, patients over the age of 50 with a GGT level of 90 IU/L or higher exhibited significantly greater improvements, indicating the potential of GGT in selecting the most suitable candidates for treatment [55].

A research study involving 125 individuals diagnosed with HIV and 59 control subjects revealed that IL-18 levels were significantly elevated in those with HIV. This increase correlated with higher liver enzyme levels (AST and ALT) and a reduced liver-to-spleen ratio, indicating a potential association between IL-18 and liver damage in NAFLD [56].

The findings of this study indicated that both IL-18 and A2M showed significantly positive correlations across all examined groups, with r values ranging from 0.662 to 0.946. While the direct interactions between IL-18 and A2M in NAFLD have not been thoroughly investigated, their involvement in inflammation and tissue remodeling may overlap. The proinflammatory IL-18-dependent signaling acts as a modulator of early liver damage in fatty liver, which occurs prior to the onset of histologic NASH [57]. The inflammation induced by IL-18 may activate proteases that A2M subsequently inhibits, thus influencing the inflammatory environment and tissue repair mechanisms in the liver [15].

### Interactions between lipids, inflammation and steatosis

#### Blood lipids, IL18 and A2M association

A negative significant correlation was observed between HDL-C and IL 18 in participants with mild fatty liver, a same significant negative relationship was also present in patients with both mild and moderate fatty livers with A2M parameters. On the contrary, VLDL-C showed a significant positive association with IL 18 only among moderate fatty liver patients. In this context, both IL-18 and A2M play roles in inflammatory pathways that can affect lipid metabolism. The relationship between IL-18 and dyslipidemia seems to be reciprocal; IL-18 not only worsens lipid irregularities, but dyslipidemia can also increase IL-18 production [58].

#### GGT, IL18 and A2M association

Additionally, a positive correlation was observed among IL-18, A2M, and GGT in individuals with moderate fatty liver. In this regard, increased serum levels of IL-18 have been linked to heightened liver enzymes and hepatic steatosis in patients with NAFLD, suggesting its potential as a biomarker for liver damage [56]. While the direct interactions between GGT and A2M have not been thoroughly investigated, their involvement in liver function and fibrosis implies a possible connection. Elevated GGT levels reflect oxidative stress and liver damage, which may trigger the activation of proteases that A2M subsequently inhibits, thus influencing the inflammatory environment and tissue repair mechanisms in the liver.

IL-18, A2M, inflammation, and fibrosis may interact through a proposed feed-forward loop during chronic tissue injury. IL-18, which is released following inflammasome activation, enhances inflammatory signaling and triggers the production of profibrotic cytokines such as TGF-β1 and IL-13. This process promotes the activation of fibroblasts and the deposition of extracellular matrix (ECM) [59]. Tissue injury and the release of proteases stimulate the liver to produce A2M, a broad-spectrum protease inhibitor that also binds to cytokines and growth factors, including TGF-β, thereby modulating their stability and bioavailability [15]. By inhibiting matrix-degrading metalloproteases, A2M may decrease ECM turnover, while its ability to bind cytokines could extend or localize profibrotic signaling. The inflammation driven by IL-18 and the modulation of protease and cytokine activity by A2M may mutually reinforce each other, leading to a shift in wound-healing responses towards chronic fibrosis [60].

#### Impact of dietary intervention on patients with MAFLD

The analysis of the participants’ daily dietary intake indicated a high consumption of most macronutrients among patients with fatty liver, particularly those with moderate disease, alongside a low daily fiber intake. Significant negative correlations were observed between both of the IL-18 and A2M, and the daily fiber and protein intakes, while a positive association was noted with total lipids and saturated fatty acids (SFAs). Furthermore, patients exhibited a low score on Günther’s DASH index.

Assessing the impact of dietary intervention on individuals with MAFLD revealed notable enhancements in liver function and metabolic indicators. Participants adhered to a well-organized nutritional regimen that prioritized decreased caloric consumption, a balanced distribution of macronutrients, and a higher intake of foods rich in fiber and antioxidants, including fruits, vegetables, whole grains and lean proteins with high Günther’s DASH index. By the end of the intervention period, the patients showed a marked reduction in hepatic steatosis as measured by improved liver GGT enzyme, IL-18 and A2M levels. Recent evidence supports the application of mediation models in the context of dietary interventions and metabolic dysfunction-associated fatty liver disease (MAFLD/MASLD). For example, a large 2025 study using NHANES data demonstrated that metabolic and inflammatory factors can significantly mediate the relationship between dietary quality and MASLD outcomes, highlighting the relevance of such approaches in nutritional research [61]. Similarly, mediation frameworks have been widely used to investigate the role of liver enzymes such as gamma-glutamyltransferase (GGT) as intermediate variables linking metabolic risk factors to clinical outcomes [62]. More recent studies have also emphasized the mediating role of inflammatory and metabolic biomarkers in the relationship between nutritional status and liver disease progression [63]. Additionally, many participants experienced beneficial changes in body weight, FBG, and lipid profiles, indicating an overall improvement in metabolic health. These findings support the role of diet as a cornerstone in the management of NAFLD and highlight the potential for lifestyle modifications to slow or even reverse the disease progression. Research involving 3051 middle-aged and older adults found that higher scores on the DASH diet were negatively correlated with the prevalence of MAFLD. Path analysis indicated that these relationships were mediated by decreases in insulin resistance, triglycerides, inflammation and BMI [64].

A culturally customized low-calorie diet significantly enhanced hepatic steatosis, inflammatory status, and antioxidant capacity in Egyptian patients with MAFLD. These enhancements were partly independent of weight and were also influenced by anti-inflammatory responses. The results endorse low-calorie dietary approaches as a potentially scalable therapeutic strategy for managing MAFLD in resource-constrained environments. However, the lack of a control group restricts causal conclusions, necessitating further assessment of implementation feasibility and cost-effectiveness. Moreover, additional advantages were noted in lifestyle behaviors, including physical activity and sleep [65].

### Strengths, limitations and future directions

This study is strengthened by its prospective interventional design, emphasis on dietary modification, and the assessment of key metabolic and inflammatory biomarkers, including IL-18 and A2M, using standardized methods. However, several limitations should be acknowledged, including the relatively small sample size, inclusion of women only, reliance on self-reported dietary data, and the observational nature of the analysis, which may limit causal inference. Although the reduction in A2M was statistically significant, its small magnitude, expected in early-stage steatosis without established fibrosis, and the absence of defined clinical thresholds warrant cautious interpretation.

Despite these limitations, the findings provide preliminary evidence supporting the interplay between metabolic dysfunction, inflammation, and liver injury in MAFLD, as well as the potential benefits of dietary interventions. Future longitudinal and mechanistic studies with larger and more diverse populations are needed to further elucidate the roles of IL-18 and A2M in MAFLD progression and to evaluate their potential as therapeutic targets.

## Conclusion

The findings of this study highlight the association of multiple metabolic disorders with the progression and severity of MAFLD. Significant elevations of IL-18 and A2M in affected individuals underscore their role in linking inflammation and metabolic imbalance. Dietary intervention improved anthropometric and liver function measures, supporting its beneficial effect in MAFLD. These results suggest that IL-18 and A2M may serve as biomarkers and potential therapeutic targets. Further studies with larger cohorts and longer follow-up are warranted to clarify their mechanistic roles and therapeutic potential.

## Data Availability

The data supporting the findings of this study are available from the corresponding author upon reasonable request, after taking the permission of our institute “National Research Centre”.
